# Silent Allies: Endophytic Entomopathogenic Fungi Promote Biological Control and Reduce Spittlebug *Mahanarva spectabilis* Distant, 1909 (Hemiptera: Cercopidae)

**DOI:** 10.3390/jof11070492

**Published:** 2025-06-27

**Authors:** Michelle O. Campagnani, Luís Augusto Calsavara, Charles Martins de Oliveira, Alexander Machado Auad

**Affiliations:** 1 Entomology Laboratory, Embrapa Gado de Leite, Juiz de Fora 36038-330, MG, Brazil; mcampaginani@gmail.com; 2 Department of Entomology and Acarology, Luiz de Queiroz College of Agriculture, University of São Paulo, Piracicaba 13418-900, SP, Brazil; agrocalsavara@gmail.com; 3 Entomology Laboratory, Embrapa Cerrados, Brasília 73310-970, DF, Brazil; charles.oliveira@embrapa.br

**Keywords:** entomopathogenic fungi, endophytic, biological control, pasture, spittlebug

## Abstract

*Urochloa ruziziensis* (R. Germ. and C.M. Evrard) Crins (synonym *Brachiaria ruziziensis*) Poales: Poaceae) pastures are often attacked by spittlebugs, compromising their biomass for livestock usage. A sustainable control method involves the use of entomopathogenic fungi. Therefore, the objective of this study was to evaluate the efficacy of controlling *Mahanarva spectabilis* Distant, 1909 (Hemiptera: Cercopidae), in greenhouse and field conditions via endophytic entomopathogenic fungi. In the greenhouse, the mortality of nymphs and adults was 100%, and more than 53% of the nymphs and 59% of the adults that fed on plants inoculated with *Fusarium multiceps* and *Metarhizium anisopliae* presented with these fungi in their cadavers. In the field, more than 45% of the insect cadavers that had fed on plants grown from fungus-treated seeds were found to contain the fungi. *F. multiceps* was found to be endophytic in more than 60% of the plants up to 90 days after seed treatment, and *M. anisopliae* was found in more than 70% of the plants up to 120 days after treatment. The damage scores of the control plants, both in the greenhouse and in the field, were greater than those of the plants inoculated with the fungi. *F. multiceps* and *M. anisopliae* in the endophytic pathway of *U. ruziziensis* are therefore efficient at controlling spittlebugs.

## 1. Introduction

Attacks from nymph and adult spittlebugs such as *Mahanarva spectabilis* (Distant, 1909) (Hemiptera: Cercopidae) damage host plants by sucking their sap and injecting toxins that induce phytotoxicity, thereby reducing photosynthetic rates and subsequently reducing the availability of biomass for livestock [1,2,3]. Brazilian cattle are raised in an extensive livestock system made up of native and cultivated species, and these pastures account for approximately 45% of the country’s agricultural area [4]. Outbreaks of spittlebugs on *Urochloa* spp. (Hochst. ex.A. Rich.) RD Webster (synonym of *Brachiaria* spp. [Hochst. ex A. Rich Stapf]) are damaging the beef and milk production chain [2,5,6].

Chemical control is insufficient, has negative environmental impacts and is economically unviable in pasture systems [7]. Furthermore, the use of these phytosanitary products contradicts the United Nations’ Sustainable Development Goals (SDGs). The SDGs are global actions to protect the environment and climate; reduce poverty; ensure that people have access to healthier food; and promote responsible consumption and production, which are some of the goals of the 2030 Agenda [8]. As such, other strategies to combat spittlebug outbreaks in a sustainable way are being researched. These include the constitutive and induced resistance of plants to spittlebug attacks [9,10,11,12], the diversification of pastures [13], the use of plant compounds as biocontrol agents [14,15], soil fertilization [16,17], the use of entomopathogenic fungi [18,19,20] and the implementation of a silvopastoral consortium in relation to monocultures, all resulting in reductions in the spittlebug population [21]. The diapause of the pasture spittlebug is a crucial factor for the survival and proliferation of this pest, and this physiological mechanism complicates pest management [22].

The action of some entomopathogenic fungi on insect pests, directly through contact or indirectly through the internal colonization of plant tissues (endophytes), can offer a control option within integrated pest management (IPM) programs [23,24]. The use of entomopathogenic fungi is a sustainable strategy, but there are challenges in its application [25]. With respect to the use of fungi, their success is affected by abiotic factors because they are more effective at mild temperatures [26]. Therefore, it is important to consider the species of entomopathogenic fungi that live as endophytes within plant tissues to mitigate the effects of these abiotic factors. Favorable results have been shown with seed treatment as a technique for introducing microorganisms into plants [3,19]. Endophytic entomopathogenic fungi are environmentally safe, can be mass produced and can infect insects at various stages of their cycles [27,28]. The use of endophytic entomopathogenic fungi, such as *Metarhizium brunneum* Petch. and *Beauveria bassiana* (Balsamo) Vuill., has gained interest as an important component of modern IPM programs [24].

The entomopathogenic fungi *Fusarium multiceps* (Hypocreales: Nectriaceae) and *Metarhizium anisopliae* (Metschn.) Sorokīn (Hypocreales: Clavicipitaceae) were previously isolated from the spittlebug *M. spectabilis* in a silvopastoral system in the state of Maranhão, Brazil [18]. Both strains can cause high insect mortality and colonize *Urochloa ruziziensis* (R. Germ. & C. M. Evrard) Crins, making the forage an efficient vector for the biological control of *M. spectabilis* via the seed inoculation technique [19,20]. Therefore, this study aimed to evaluate the efficacy of *F. multiceps* and *M. anisopliae* in controlling the adults and nymphs of *M. spectabilis* in greenhouse and field conditions, as well as to confirm the persistence of these fungi in plant tissues in both environments.

## 2. Materials and Methods

The experiments were conducted in a greenhouse, at the Entomology Laboratory and in an experimental field (Embrapa Gado de Leite), José Henrique Bruschi, Coronel Pacheco city (21°33′23.7″ S 43°16′09.2″ W), Minas Gerais State, Brazil.

### 2.1. Obtaining Fungi and Urochloa ruziziensis Seeds

The fungal strains *F. multiceps* and *M. anisopliae*, which were isolated from naturally infected pasture spittlebugs, *M. spectabilis*, were collected at Fazenda Monaliza in the western state of Maranhão (Brazil), at coordinates 05.5255556°, 47.4430556°. The farm consists of pastures of *Urochloa brizantha* [(Hochst. ex A. Rich.) Stapf] (Poaceae) cultivar ‘Marundu’ managed in a silvopastoral system with the natural regeneration of native shrubs and trees since 1998. The two strains of entomopathogenic fungi used in these experiments were those deposited in the Microorganism Collection of the Federal University of Minas Gerais, Brazil (CM-UFMG; World Data Center for Microorganisms [WDCM] 1029), with the following accession numbers: *Fusarium multiceps* UFMGCB 11443 (GenBank: ON831395) and *Metarhizium anisopliae* UFMGCB 11444 (GenBank: ON831396). Their DNA sequences were deposited in the GenBank sequence database via the BLASTn program (version 2.215) from the National Center for Biotechnology Information, which is available on its website. *U. ruziziensis* cv. Kennedy seeds were purchased from SOESP (Presidente Prudente, São Paulo, Brazil).

### 2.2. Seed Treatment for Greenhouse and Field Bioassays

For each fungal isolate, 100 g of seeds was used in the greenhouse bioassays and 300 g was used in the field experiments. The *U. ruziziensis* seeds were first weighed and then surface-disinfected by washing them with 2% sodium hypochlorite for two minutes, followed by washing with 70% ethanol for one minute. Afterwards, the seeds were rinsed with sterile distilled water, following a protocol adapted from Carvalho et al. [29] and Ferreira et al. [30], and then prepared for fungal inoculation.

### 2.3. Inoculation of Fungi in Seeds

The *F. multiceps* and *M. anisopliae* isolates were reactivated and prepared separately to produce conidia. These were grown in 9.0 cm × 1.5 cm Petri dishes containing a Sabouraud dextrose agar (Kasvi**^®^**, Lehigh County, PA, USA) culture medium (4% dextrose, 1% casein and 1.5% agar; Kasvi**^®^**, Lehigh County, PA, USA), which was poured into a vertical laminar flow chamber previously sterilized with 70% alcohol. These plates were incubated in a biochemical oxygen demand (BOD) EletroLab**^®^** (Campinas, Brazil) at 25 ± 2 °C and 70 ± 10% RH with a 12 h photoperiod for vegetative growth and conidiogenesis. After incubation for approximately 7 days, the conidia produced were removed from the surface of the culture medium with a sterile metal spatula and inoculated separately to prepare suspensions containing sterile distilled water and an ionic surfactant (Tween 80 [0.01%], Sigma-Aldrich**^®^**, Steinheim, Germany) at a concentration of 1 × 10^8^ conidia/mL. The suspensions obtained were stirred via a magnetic stirrer, and the concentrations were estimated via a hemocytometer.

Seed treatment was performed by inoculating 100 g of seeds in 200 mL of sterile distilled water containing 0.01% Tween 80 as a surfactant. Conidia of *M. anisopliae* and *F. multiceps* were added to the solution until they reached a concentration of 1 × 10^8^ conidia/mL. As a control, a solution of 200 mL of sterile distilled water with 0.01% Tween 80 and without fungal conidia was used to treat the seeds. This setup resulted in three treatments: plants from seeds treated with *F. multiceps*; plants from seeds treated with *M. anisopliae*; and control plants, whose seeds were not treated. The seeds remained in contact with the suspension for 60 min, following the methodology adapted from Keyser et al. [31]. The viability of the conidial batches was tested before the experiments according to the method described by Lopes et al. [32].

### 2.4. Cultivation of Urochloa ruziziensis Containing Entomopathogenic Fungi in the Greenhouse

Immediately after being treated, ten *U. ruziziensis* seeds, treated with or without entomopathogenic fungi, were placed in plastic pots with a capacity of 1 kg, which contained a commercial substrate (Carolina Soil**^®^**—Santa Cruz do Sul, Brazil—composition: sphagnum turf, expanded vermiculite, dolomitic calcareum, agricultural gypsum and NPK fertilizer). The plants were automatically irrigated three times a day for 15 min throughout the experiment. After 45 days, the first pruning was carried out, and the plants were cut to a height of 15 cm. The insect tests were conducted 90 days after the seeds were planted.

### 2.5. Obtaining the Insect Pest Mahanarva spectabilis

Adults and nymphs of *M. spectabilis* were collected manually from clumps of elephant grass in the experimental field of Embrapa Dairy Cattle (21°33′23.7″ S 43°16′09.2″ W—Coronel Pacheco—Minas Gerais, Brazil). An average of 1000 nymphs and 500 adults were collected between 9 a.m. and 12 p.m. The adults were placed in 30 cm × 30 cm × 60 cm (width × depth × height) entomological cages, and the nymphs were placed in 5 cm × 15 cm (diameter × height) plastic pots containing elephant grass plants. The insects were taken to the Entomology Laboratory at Embrapa in Juiz de Fora.

### 2.6. Bioassays in the Greenhouse

#### 2.6.1. Control of *Mahanarva spectabilis* Nymphs and Adults with Endophytic Fungi

A randomized block design (RBD) was adopted with the three treatments (plants from seeds treated with *F. multiceps*; plants from seeds treated with *M. anisopliae*; and control plants, whose seeds were not treated with fungi). A total of 10 third- or fourth-instar *M. spectabilis* nymphs were used per plant, with 20 replicates per treatment, totaling 600 insects and 60 experimental units (three treatments × 20 replicates × 10 insects). The pots were individually covered in voile bags to avoid cross-contamination between the treatments and insect escape. Twenty-four hours after release, insect mortality was assessed daily for six days. Dead nymphs were collected daily and placed in 1.5 mL microcentrifuge tubes for subsequent analysis of the cause of death.

For the adult trial, five pairs were placed in pots in the greenhouse at Embrapa Dairy Cattle, totaling 270 insects and 27 experimental units (3 treatments × 9 replicates × 10 insects). These pots were placed inside entomological cages [60 cm × 35 cm × 25 cm (height × width × diameter)] to prevent cross-contamination between treatments and insect escape. Twenty-four hours after release, adult mortality was assessed daily for nine days. Dead adults were collected daily and placed in 1.5 mL microcentrifuge tubes for subsequent analysis of the cause of death.

#### 2.6.2. Evaluation of Persistence of Fungi in Nymph and Adult Corpses in the Greenhouse

Subsequently, surface disinfestation was performed on the cadaver samples of the nymphs and adults, and each sample was treated individually by washing it in 2% hypochlorite for two minutes, followed by washing in 70% alcohol for one minute. After these steps, the insects were rinsed with sterile distilled water, according to the methodology adapted from Carvalho et al. [29] and Ferreira et al. [30], and placed in Petri dishes containing a Sabouraud dextrose agar (Kasvi**^®^**, Lehigh County, PA, USA) culture medium. The plates were incubated at 25 ± 2 °C for approximately 7 days in a BOD chamber until fungal sporulation occurred, and the cause of death was confirmed according to the methodology adapted from Klieber and Reineke [33] and Ahmad et al. [34] to verify the presence of the fungi in the cadavers.

#### 2.6.3. Damage Score of the Plants

The visual damage to the leaf area of each plant subjected to attack by nymphs or adults was assessed as a percentage by three assessors, and the average was converted into a damage score from 1 to 5 [35], with 1 being the least damaging and 5 the most damaging. After analysis, the average damage to the forage was classified on the basis of the average score, adapted from Pabón et al. [36].

#### 2.6.4. Confirming Persistence of Fungi in Plant Tissues

Samples of plant tissue (leaves) were taken at random points on the plants, totaling 60 leaf samples (1 sample × 3 treatments × 20 replicates) at 30, 60, 90, 120, 150, 180 and 210 days after sowing. These leaves were packed in sterile plastic and transported to the Entomology Laboratory at Embrapa Dairy Cattle. Each leaf was superficially disinfected by immersion in 70% ethanol (1 min) and 2% sodium hypochlorite (1 min), followed by washing with sterile distilled water (2 min) [29]. After disinfestation, two fragments of each leaf were placed in Petri dishes containing a Sabouraud dextrose agar (Kasvi**^®^**, Lehigh County, PA, USA) culture medium. The plates were incubated at 25 ± 2 °C for approximately 7 days in a BOD climate chamber. To verify the presence of the inoculated fungi, the macromorphology and micromorphology of the fungi present on the plant fragments were compared with those of *F. multiceps* or *M. anisopliae* according to a methodology adapted from [37]. If one of the leaf replicates obtained from a single plant showed fungal growth characteristics of the applied fungi, it was classified as being endophytically colonized according to the methodology adapted from Klieber and Reineke [33]. Endophytic confirmation was adopted when the majority (more than 60%) of the samples presented the characteristics of the applied fungi.

### 2.7. Bioassay in the Field: Control of Mahanarva spectabilis Adults with Endophytic Fungi

The trials were conducted at the experimental field of Embrapa Dairy Cattle, José Henrique Bruschi, Coronel Pacheco, MG.

#### 2.7.1. Cultivation of *Urochloa ruziziensis* Containing Entomopathogenic Fungi in the Field

**Figure 1:**
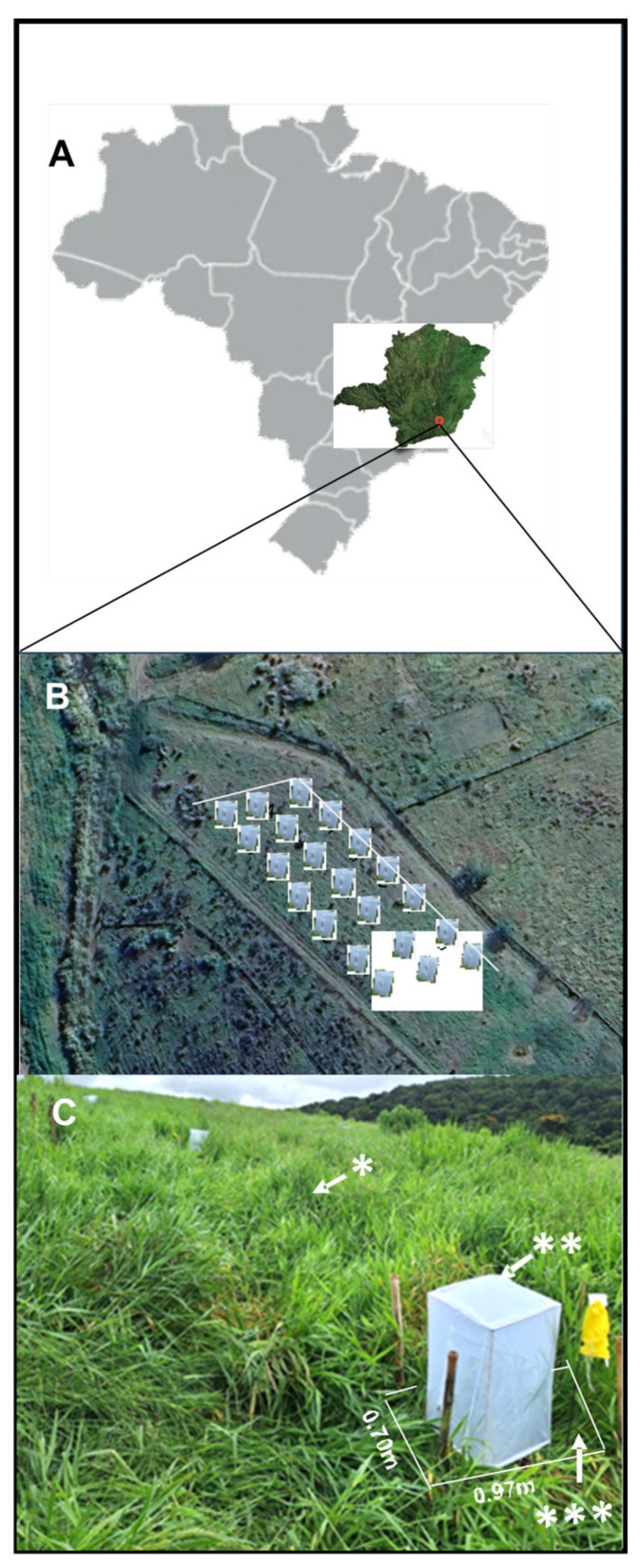
The field bioassay. The location of the experimental area in the state of Minas Gerais, Brazil, South America (**A**); the distribution of cages in the experimental area (**B**); and a detailed view of the experimental unit area with the cage (**C**). The annotations indicate the following: * natural occurrence in the plants more than 5 years ago without any treatment with a fungus; ** artificial infestation of plants treated with the fungi *Fusarium multiceps* or *Metarhizium anisopliae* or untreated; *** natural occurrence in the treated plants.

Plants from seeds treated with *F. multiceps*, plants from seeds treated with *M. anisopliae* and control plants (in which the seeds were not treated with fungi) were placed in plastic trays measuring 0.97 m × 0.70 m (length × width) and filled with soil, sand and manure in a 3:1:1 ratio [1,2,3]. The plants were automatically watered three times a day for 15 min. The plants were placed in a greenhouse and fertilized every 30 days with 30 mL of an NPK suspension (150 g of urea, 60 g of potassium chloride, 60 g of simple superphosphate, and 10 L of water). After 30 days, the first pruning was carried out, and the plants were cut to a height of 15 cm. These plants, which were kept in trays, were taken to the field 60 days after planting, maintained for 15 days for adaptation and then planted in the soil in an area of monocultured *U. ruziziensis* with a history of pasture spittlebugs for 5 consecutive years. The total area of *U. ruziziensis* was 80 m × 30 m (length × width). The plants from seeds treated with *F. multiceps*, plants from seeds treated with *M. anisopliae* and control plants were arranged in plots 10 m wide × 10 m long (Figure 1). The experimental design was a randomized block design with three treatments and seven replications, totaling twenty-one experimental units (EUs).

#### 2.7.2. Artificial Infestation of Plants by Spittlebugs

Adults and nymphs of *M. spectabilis* were manually collected from clumps of elephant grass. An average of 500 adults and 500 nymphs were collected between 8 am and 11 am. Immediately after collection, the insects were separated and placed in each EU, which contained the plants introduced into the area, accounting for approximately 50% of the introduced plants, for a total of 21 cages (Figure 1). The artificial infestation with the insects selected in the field was carried out with 630 samples (15 nymphs plus 15 adults = 30 insects/cage). The 21 cages (3 treatments and 7 replications) were observed weekly. The dead nymphs and adults were removed and placed in 1.5 mL microcentrifuge tubes for subsequent analysis of the cause of death. Insect mortality was assessed for 30 days.

#### 2.7.3. Natural Occurrence of Adults

The experimental units, which consisted of plants introduced into the area with seeds treated with *F. multiceps* or *M. anisopliae*, were transplanted into plots measuring 1.0 × 0.6 m. Within each plot, some of the plants were enclosed by a cage (0.45 × 0.80 m) to create an artificial environment. Observations were also made of the external area around the cage within the transplanted perimeter and of plants over five years old without any treatment (total experimental area). These plants were observed weekly between November 2023 and April 2024 to assess the interaction of spittlebugs with plants treated with entomopathogenic fungi. The insects were counted via an entomological sweep net, transects that covered the entire experimental area for 30 min/day of sampling were constructed and fluctuations in the adult spittlebug population were determined. These samples were taken on a weekly basis after the introduction of the fungus-treated plants to the area for three months (Figure 1).

#### 2.7.4. Evaluation of Persistence of Fungi in Nymph and Adult Corpses in the Field

From each treatment under natural or artificial infestation, five samples of nymphs or adult spittlebugs were randomly selected each week and placed in sterile centrifuge microtubes for analysis of the cause of death. These samples were taken to the Entomology Laboratory at Embrapa Dairy Cattle. The cadaver samples were then superficially disinfected as previously described in Section 2.6.2 and placed in Petri dishes containing a sterile Sabouraud dextrose agar (Kasvi**^®^**, Lehigh County, PA, USA) medium. The cadavers were inoculated in the culture medium to verify the presence of the fungi *F. multiceps* and *M. anisopliae*. These samples were incubated at 25 ± 2 °C for approximately 7 days in a climate-controlled BOD chamber until fungal sporulation occurred, after which their ability to cause mortality was assessed. The samples that did not show fungal growth were considered to have died from natural causes.

#### 2.7.5. Damage Score

For the plants subjected to natural or artificial infestation, visual damage to the leaf area of each plant was assessed as a percentage by three evaluators. The average was converted into a damage score and analyzed as described in Section 2.6.3.

#### 2.7.6. Confirming the Persistence of Fungi in the Plant Tissue of *Urochloa ruziziensis*

Samples were taken at random from the plants introduced into the experimental area at the Entomology Laboratory of Embrapa Dairy Cattle. A total of 3 leaves were collected at random points from each introduced plant, totaling 21 leaf samples [3 leaves × 7 replicates (*F. multiceps*, *M. anisopliae*, and control)] per sampling event. These leaves were placed in sterile plastic bags and taken to the laboratory for testing. Each leaf was processed as described in Section 2.6.4. This assessment was conducted 30 days after the fungus-treated seeds were planted to determine whether the fungi persisted in the plant tissue over time. Further evaluations were made at the first pruning of the introduced plants and after 60, 90, 120, 150, 180 and 210 days.

### 2.8. Statistical Analysis

All data analyses were performed via R version 4.2.2 [38]. Initially, the data were assessed for normality via the Shapiro–Wilk test and for homogeneity of variance via Levene’s test. For variables showing a normal distribution and homogeneity of variance, one-way ANOVA was applied, with mean comparisons conducted using Tukey’s test (*p* < 0.05). For variables that did not meet the assumptions of normality or homogeneity, generalized linear models (GLMs) were fitted using Poisson or binomial distributions. When overdispersion was detected in the Poisson models, a quasi-Poisson approach was used to estimate the dispersion parameter. Model significance was assessed via the ANOVA function in R, and models were fitted via log or logit link functions as appropriate. In experiments with repeated measurements over time, generalized linear mixed models (GLMMs) were applied to account for the hierarchical data structure, with the treatment (fungi and control) as a fixed effect and date as a random effect. Analyses were performed via the “car” [39], “emmeans” [40], “lme4” [41], “MASS” [42]) and “multcomp” [43] packages. All tests were conducted at the 5% significance level (*p* < 0.05).

## 3. Results

### 3.1. Mortality of Mahanarva spectabilis Nymphs and Adults Caused by the Fungi Fusarium multiceps and Metarhizium anisopliae in a Greenhouse

**Figure 2:**
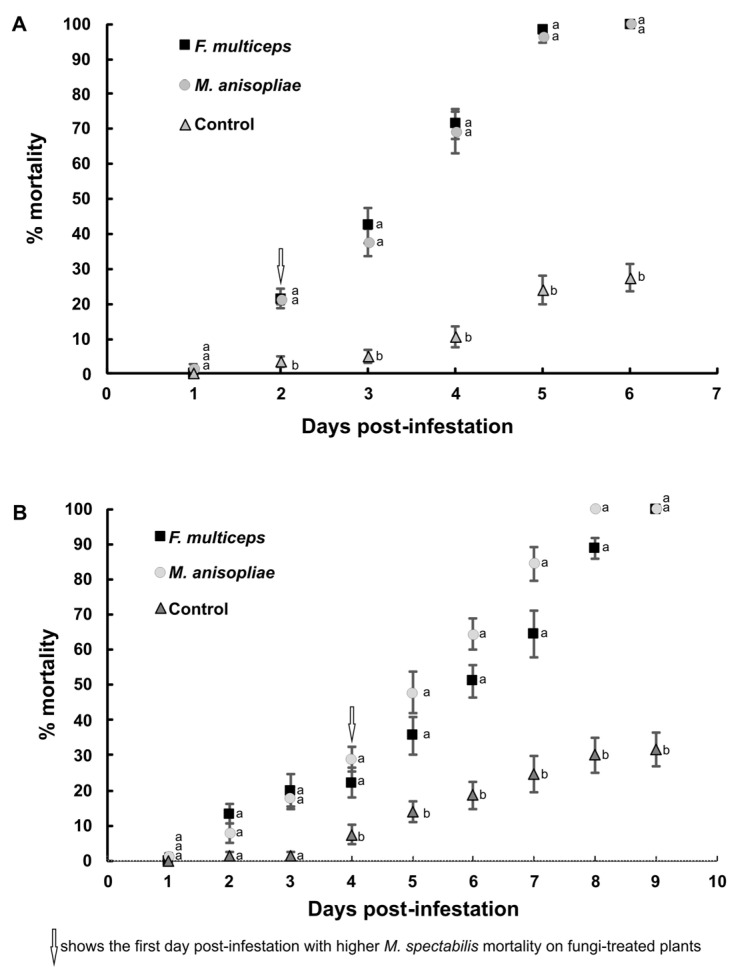
The mortality cumulative (means ± SE) of *Mahanarva spectabilis* nymphs (**A**) and adults (**B**) that fed on plants treated with the fungi *Fusarium multiceps* or *Metarhizium anisopliae*, or the control. Cumulative means followed by different letters on the same day post-infestation differ significantly according to Tukey’s test (*p* < 0.05).

In the plants grown from untreated seeds, the mortality of nymphs (X^2^_2;354_ = 495.49; *p* < 0.0001) and adults (X^2^_2;337_ = 178.82; *p* < 0.0001) was significantly lower than of the plants grown from seeds treated with fungi (Figure 2A,B). From the second day of infestation onwards, nymph mortality was significantly greater on the plants originating from seeds treated with *M. anisopliae* or *F. multiceps* (Figure 2A). By the sixth day of the experiment, 100% mortality of *M. spectabilis* nymphs that fed on *U. ruziziensis* from fungus-treated seeds was observed.

Starting from the fourth day of infestation, adult mortality was also significantly greater when the nymphs fed on fungus-treated plants (Figure 2B). By the ninth day of infestation, both *M. anisopliae* and *F. multiceps* induced 100% mortality in *M. spectabilis* adults.

### 3.2. Mortality and Population Fluctuations of Mahanarva spectabilis Nymphs and Adults During Artificial and Natural Infestations in the Field

The mortality of insects that fed on plants introduced into the field and confined within cages (artificial infestation) was 4.7 times greater for seeds treated with *Fusarium multiceps* and *Metarhizium anisopliae* (267 insects) than for those with untreated seeds (57 insects), with significant differences observed for both nymphs (X^2^_2;198_ = 6.64; *p* = 0.036) and adults (X^2^_2;198_ = 6.20; *p* < 0.044).

**Figure 3:**
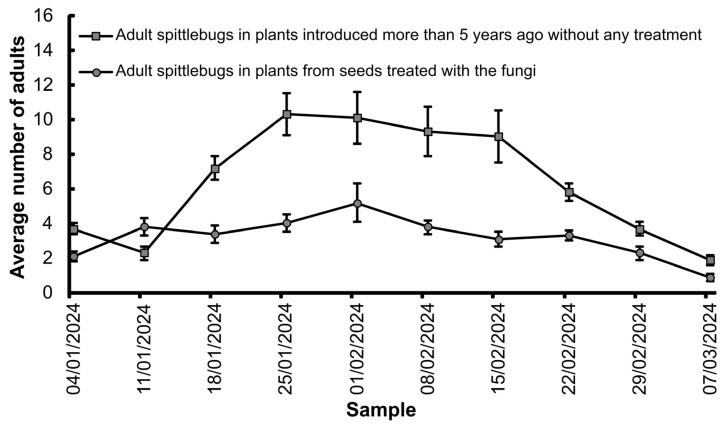
The population fluctuations (means ± SE) of spittlebug adults in the field experiment, comparing plants that had been established in the field for 5 years and had not received any type of treatment with the plants that were newly introduced into the area and originated from seeds treated with the fungus *Fusarium multiceps* or *Metarhizium anisopliae*.

In the case of natural infestations, there was a significant difference in the occurrence of nymphs (X^2^_2;63_ = 10.81; *p* < 0.0001) and adults (X^2^_2;120_ = 34.57; *p* < 0.0001) on plants introduced into the field outside the cages, with 75% of all the sampled insects (353 insects) found in the control treatment. The population of adult spittlebugs in the experimental field area fluctuated over time and was significantly greater (X^2^_1;189_ = 100.08; *p* < 0.0001) on the plants that had been established in the field for five years without seed treatment than on the plants introduced into the area from seeds treated with *F. multiceps* or *M. anisopliae* (Figure 3).

### 3.3. Confirmation of the Presence of Fungi in Nymphs and Adults of Mahanarva spectabilis Killed While Feeding on Different Plants in the Greenhouse and Field

The number of dead insects with fungi present in their tissues differed significantly between the fungus-treated and control treatments for both nymphs (X^2^_2;15_ = 227.19; *p* = 0.008) and adults (X^2^_2;24_ = 183.75; *p* = 0.020). Fungi were detected in 53% of the nymphs killed by *Fusarium multiceps* and *Metarhizium anisopliae*, and in 78% and 59% of the adults, respectively (Table 1). No fungi with macro- or micromorphological characteristics similar to those of *F. multiceps* or *M. anisopliae* were isolated from the control treatment. Additionally, more than 45% of the insects that died after being artificially introduced into or naturally infesting the field presented with the respective fungi, confirming their role in insect mortality (Table 1). Likewise, no similar fungi were isolated from the control insects (Table 1).

### 3.4. Confirmation of the Presence of Fungi in Plant Tissues Under Greenhouse and Field Conditions

In both the greenhouse and field bioassays, the entomopathogenic fungi *F. multiceps* and *M. anisopliae* were isolated from *U. ruziziensis* plant tissues at 30, 60, 120, 150, 180 and 210 days after seed treatment, confirming their endophytic persistence. No fungi with similar characteristics were found in the plants grown from untreated seeds (controls) in any of the bioassays. In the greenhouse, 88.9% of the plants evaluated 30 days after sowing presented with *M. anisopliae* in their tissues, whereas 77.8% presented with *F. multiceps*, which was significantly greater than the percentage in the untreated control (X^2^_2;159_ = 7.60; *p* = 0.023) (Table 2). *M. anisopliae* remained in more than 60% of the plant tissue samples for up to 120 days, whereas *F. multiceps* persisted for up to 90 days (Table 3). In the field, 85.7% of the plants presented the fungi at 30 days after sowing, which was significantly greater than the percentage in the control (X^2^_2;119_ = 6.66; *p* = 0.036). Both fungi persisted in more than 60% of the plant samples up to 120 days post-treatment (Table 2).

### 3.5. Leaf Damage Caused by Mahanarva spectabilis Nymphs and Adults in the Greenhouse and Field

Under greenhouse conditions, the control plants presented significantly greater damage from *M. spectabilis* nymphs (X^2^_2;57_ = 23.24; *p* < 0.0001) and adults (F_2;24_ = 13.65; *p* = 0.0001) than the plants grown from seeds treated with *F. multiceps* and *M. anisopliae*. Similarly, in the field, adult spittlebugs caused significantly more damage to the control plants than to those treated with the fungi (X^2^ = 22.09; *p* < 0.0001) (Table 3).

## 4. Discussion

This study evaluated the potential of two species of endophytic entomopathogenic fungi, *F. multiceps* and *M. anisopliae*, in the management of *M. spectabilis* populations, promoting the effective control of spittlebugs through the treatment of *U. ruziziensis* seeds. The fungi *F. multiceps* and *M. anisopliae* showed the ability to reduce the population of nymphs and adults of *M. spectabilis* in greenhouse and field experiments. These fungi are therefore promising bioinputs for the management of spittlebugs.

Entomopathogenic fungi are microorganisms with great potential for use in the biological control of insect pests because of their various modes of action [44]. As demonstrated in this study, entomopathogenic fungi can exert biocontrol effects on pest insects after they colonize plants as endophytes [45]. The fungi of the *Metarhizium* species are promising bioinputs essential for sustainability and innovation in pest management [46,47,48].

In Brazil, there are ongoing studies investigating the use of endophytic entomopathogenic fungi in the management of spittlebugs in pastures [18,19,20]. This study revealed 100% mortality of *M. spectabilis* spittlebugs when the endophytic pathway via *U. ruziziensis* was used to induce epizootics in the insects. In contrast, Macedo et al. [49] reported mortality rates ranging from 10.5 to 60% when *M. anisopliae* was sprayed on *Mahanarva fimbriolata* (Stal, 1854) (Hemiptera: Cercopidae) nymphs. Native isolates of *M. anisopliae* have been shown to control 20–50% of spittlebug populations through the inundative biological control method in sugarcane [50]. Loureiro et al. [51] reported that most *M. anisopliae* isolates caused 41–50% mortality by the fourth day after being sprayed on *M. fimbriolata*, with mortality increasing to 81–90% by the sixth day. Other studies conducted by Campagnani et al. [20] in a greenhouse used the foliar spraying of *F. multiceps* and *M. anisopliae* and reported mortality rates over 90% against *M. spectabilis*. Mateus et al. [52] tested various registered *M. anisopliae*-based bioinsecticides and reported 80% mortality in *M. fimbriolata* nymphs.

The greater efficiency observed in the present study may be attributed to the dual role of the fungi used, which are not only entomopathogenic but also confirmed as endophytes. This condition likely protects them from the adverse abiotic factors typically affecting fungi that are applied via spraying, thereby helping to preserve their virulence—one of the key determinants of entomopathogenic efficacy.

The colonization of most *U. ruziziensis* plants by *F. multiceps* and *M. anisopliae* had an antagonistic effect on insects. Although *F. multiceps* and *M. anisopliae* caused 100% mortality in the greenhouse experiments, to assess the effectiveness of these fungi, nymph and adult corpses should be considered only if they display fungal growth with macro- and micromorphological characteristics typical of the isolates used for seed or plant treatment. *Mahanarva spectabilis* spittlebugs exhibited high mortality when they fed on *U. ruziziensis* grown from seeds treated with these fungi; among the nymphs and adults collected, sporulation was observed in more than 53% of the nymphs and 59% of the adults in the greenhouse. In the field-collected insects, both inside and outside the cages, more than 45% of the recovered corpses showed the sporulation of the fungi used in the seed treatment. As in this study, the fungal infection of insect corpses that fed on plant tissues colonized by entomopathogenic fungi has also been reported in other pest suppression studies [19,20,33,53].

In this work, the presence of fungi recovered from *M. spectabilis* cadavers, which included the characteristic mycelia of *F. multiceps* and *M. anisopliae*, suggests that the seed inoculation technique used resulted in good distribution and colonization within the plants, both in the greenhouse and in the field, such that insects ingested the fungi when feeding on the plants, which likely caused epizootics. Even so, some dead insects did not exhibit fungal growth after incubation in Sabouraud; these insects were likely infected despite the limited growth of the fungus because it produced toxins in sufficient quantities to cause their death or because the profuse growth of the fungus in the hemolymph caused homeostasis and starvation, leading to the death of the insect [54]. Consistent with the results obtained in this study, Quesada-Moraga et al. [55] reported that biological mycoinsecticides can be used with contact action or as endophytes that reach the internal tissues of host plants, negatively affecting the survival and development of pests.

In addition to their capacity for biological control, the *F. multiceps* and *M. anisopliae* strains have demonstrated the ability to colonize *U. ruziziensis*. This efficiency of the colonization of fungal strains in host plants via the endophytic route is essential to guarantee the exposure of *M. spectabilis* to entomopathogenic fungi in crops. This is an important advantage for control strategies according to Vega et al. [56] and Quesada-Moraga and Vey [57] since endophytic entomopathogenic fungi persist better in plant tissue than on the plant surface, as previously reported. Some species of endophytic fungi promote systemic protection against insect pests due to chemical alterations induced in the plant by the microorganism or by the secondary metabolites secreted by the fungus [58,59]. Interestingly, Amandio et al. [60] reported that fungal structures of *Metarhizium* were also isolated from the stems of *U. brizantha* plants after seed germination. Consistent with the findings of this study, *Metarhizium* spp. have been reisolated from plants such as sugarcane [50] and *U. brizantha* [60], and *F. multiceps* and *M. anisopliae* from *U. brizantha* [20] and *U. ruziziensis* [19]. Several genera of endophytic fungi have also been isolated from coffee tissues, including some entomopathogenic species such as *B. bassiana*, which suppresses the coffee berry borer [55]. Three entomopathogenic fungi, *B. bassiana*, *Lecanicillium lecanii* (Zimmerm.) and *Aspergillus parasiticus* (Spear), have endophytically colonized a variety of crop plants, significantly affecting herbivore performance. However, determining the mechanisms underlying these negative effects requires further investigation. Several fungi produce metabolites that may be active against insects [57].

This study revealed that both *F. multiceps* and *M. anisopliae* can colonize plants through seed treatment and persist in the majority of the phyllosphere (more than 60%) of *U. ruriziensis* for approximately 120 days in the field and in the greenhouse (Table 2). These results corroborate those of de Oliveira Netto et al. [19] who also reported the persistence of these *F. multiceps* and *M. anisopliae* fungi in more than 60% of *U. ruriziensis* plants up to 150 days after their seeds were inoculated. These results could protect the plants from spittlebug attacks for a period of 120 to 150 days, as the fungi are harbored within the tissues of *U. ruriziensis*. The data from these studies incorporate a range of published works analyzing the presence of endophytes but only for a short period in various cultivated crops [60,61,62].

Thus, the results obtained reinforce the potential of *F. multiceps* and *M. anisopliae* strains as biological control agents since the treated plants presented lower damage scores, reflecting a reduced insect infestation density. These findings demonstrate the effectiveness of using endophytic fungi as a complementary strategy for the management of *M. spectabilis* in *U. ruziziensis*.

## 5. Conclusions

The results of this study demonstrate that the fungi *F. multiceps* and *M. anisopliae* are effective in controlling *M. spectabilis* nymphs and adults in *U. ruziziensis* under both greenhouse and field conditions. Given their ability for endophytic colonization and persistence in plant tissues, these fungal strains show promise as candidates for the development of bioproducts aimed at integrated pest management.

## Figures and Tables

**Table 1:** The percentage (means ± SE) of dead insects with fungi in their tissues that fed on *Urochloa ruziziensis* plants derived from seeds treated or not treated with *Fusarium multiceps* or *Metarhizium anisopliae* in the greenhouse and field bioassays during either artificial or natural infestation.

Location	Insect Stage	Treatments	Average (%)	
Greenhouse	Nymphs	*Fusarium multiceps*	53.3 ± 11.2 ^a^	χ^2^ = 227.19; *p* = 0.008
		*Metarhizium anisopliae*	52.9 ± 11.1 ^a^	
		Control	0 ^b^	
	Adults	*Fusarium multiceps*	77.7 ± 6.0 ^a^	χ^2^ = 183.75; *p* = 0.020
		*Metarhizium anisopliae*	58.9 ± 11.7 ^a^	
		Control	0 ^b^	
Field: Artificial Infestation (*)	Nymphs	*Fusarium multiceps*	90.5 ± 6.1 ^a^	X^2^_2;11_ = 65.70; *p* < 0.0001
		*Metarhizium anisopliae*	100 ± 0.0 ^a^	
		Control	0 ^b^	
	Adults	*Fusarium multiceps*	48.8 ± 5.2 ^a^	X^2^_2;131_ = 10.18; *p* = 0.0062
		*Metarhizium anisopliae*	44.6 ± 4.9 ^a^	
		Control	0 ^b^	
Field: Natural Infestation (**)	Nymphs	*Fusarium multiceps*	51.9 ± 7.4 ^a^	X^2^_2;59_ = 9.71; *p* = 0.008
		*Metarhizium anisopliae*	47.7 ± 6.8 ^a^	
		Control	0 ^b^	
	Adults	*Fusarium multiceps*	57.5 ± 5.3 ^a^	X^2^_2;111_ = 21.63; *p* < 0.0001
		*Metarhizium anisopliae*	75.3 ± 4.6 ^a^	
		Control	0 ^b^	

* Artificial infestation refers to plants introduced into the cages with or without seed treatment. ** Natural infestation includes plants introduced outside cages recently with or without seed treatment, as well as plants established more than 5 years ago without any treatment. Means with distinct letters in the columns, for each insect stage in each location separately, indicate significant differences between the percentages of dead insects with fungi in their tissues according to Tukey’s test (*p* < 0.05).

**Table 2:** The percentage (means ± SE) of fungi present on the leaves of *Urochloa ruziziensis* plants grown from seeds treated or not treated with *Fusarium multiceps* or *Metarhizium anisopliae* over a period of 7 months in the greenhouse and in the field.

Location	Treatment	Days After Treatment						
		30	60	90	120	150	180	210
	*F. multiceps*	77.8 ± 4.7 ^a^	77.8 ± 14.7 ^a^	66.7 ± 16.7 ^a^	44.4 ± 17.6 ^a^	33 ± 16.7 ^a^	22 ± 14.7 ^a^	11 ± 11.1 ^a^
Greenhouse	*M. anisopliae*	88.9 ± 11.1 ^a^	88.9 ± 11.1 ^a^	88.9 ± 11.1 ^a^	77.8 ±14.7 ^b^	33 ± 16.7 ^a^	11 ± 11.1 ^a^	11 ±11.1 ^a^
	Control	0 ^b^	0 ^b^	0 ^b^	0 ^a^	0 ^a^	0 ^a^	0 ^a^
	*F. multiceps*	85.7 ± 14.3 ^a^	71.4 ± 18.4 ^a^	85.7 ± 14.3 ^a^	85.7 ± 14.3 ^a^	28.6 ± 18.4 ^a^	28.6 ± 18.4 ^a^	14.3 ± 14.3 ^a^
Field	*M. anisopliae*	78.5 ± 14.3 ^a^	78.4 ± 18.4 ^a^	78.4 ± 14.3 ^a^	64.3 ± 18.4 ^a^	43.8 ± 20.2 ^a^	28.6 ± 18.4 ^a^	28.6 ± 18.4 ^a^
	Control	0 ^b^	0 ^b^	0 ^b^	0 ^b^	0 ^a^	0 ^a^	0 ^a^

Means with distinct letters in the columns for each plant age indicate significant differences between the percentages of fungal presence according to Tukey’s test (*p* < 0.05).

**Table 3:** The damage scores (means ± SE) for *Urochloa ruziziensis* as a result of *Mahanarva spectabilis* nymph and adult infestations.

Location	Insect Stage	Treatment	Damage Score (%)	
Greenhouse		*F. multiceps*	46.8 ± 4.5 ^a^	
	Nymphs	*M. anisopliae*	19.5 ± 4.4 ^b^	X^2^_2;24_ = 23.24; *p* < 0.0001
		Control	76.3 ± 2.1 ^c^	
		*F. multiceps*	48.3 ± 3.1 ^a^	
	Adults	*M. anisopliae*	46.3 ± 1.3 ^a^	X^2^_2;24_ = 13.65; *p* < 0.0001
		Control	71.1 ± 3.1 ^b^	
		*F. multiceps*	46.7 ± 4.6 ^a^	
Field		*M. anisopliae*	62.5 ± 3.2 ^b^	X^2^_2;54_ = 22.09; *p* < 0.0001
		Control	71.9 ± 3.7 ^c^	

Means with distinct letters in the columns, for each insect stage in each location separately, indicate significant differences between the damage scores according to Tukey’s test (*p* < 0.05).

## Data Availability

The datasets generated or evaluated during this study are available from the first author upon reasonable request.
